# The Treatment of Abdominal Wounds: The Contrasts of Two Campaigns

**Published:** 1916-01-08

**Authors:** 


### THE TREATMENT OF ABDOMINAL WOUNDS.

### The Contrasts of Two Campaigns.

In the South African campaign the testimony of all the most credible and expert surgeons was unanimous as to the best treatment for gunshot wounds of the abdomen. Their practice was to disturb the patient as little as possible (it was even advised that the ideal plan was to leave him for some days exactly where he lay on the battlefield, merely building an arbour or tent over him to shield him from meteorological vicissitudes); to give him no food for several days, and no drink but water, very sparingly at first; to alleviate his miserable plight by the judicious use of morphine; and to treat shock by liberal doses of strychnine. Even under this *régime* the results were very unsatisfactory; but the results of immediate surgical exploration at the first opportunity were so appalling that the expectant method seemed far preferable. Acute observers—Sir George Makins, for instance*—perceived that the proof of a contention much advanced—namely, that a certain percentage of intestinal perforations would heal spontaneously and the patients recover on the rest and starvation treatment—was most exiguous; and that it was quite possible that the recoveries were seen almost wholly when the wound, though apparently traversing the abdomen, had never really done so at all, or when a projectile had actually passed through the abdomen without injuring any hollow viscus or any important blood-vessel. The authority just quoted had also sufficient penetration to express the opinion that the disappointing results of operative treatment were possibly due to the adverse conditions of veld hospitals for abdominal work, and that if operating-rooms as well equipped as those of civilian hospitals in civilised countries had been available, the results of surgical interference might have been much more favourable.

### The Fallacy of South African Experience.

Be that as it may, the medical profession at large held the conviction at the outbreak of this war that the proper policy for abdominal wounds was that of non-interference. In six months at the Front the writer never saw a single abdominal wound operated on, nor heard anyone question the validity of the time-honoured treatment. In one case (after the battle of the Marne) he met with an officer who carried it out with extraordinary thoroughness. Left behind with a big batch of wounded in a small French village with instructions to evacuate them to the base at the earliest opportunity, this officer took the law into his own hands and declined to permit any of the abdominal cases to be moved. He evacuated the other wounded, and stayed on with nine abdominal cases in the village. Meeting him later on, inquiry was made as to the results; it appeared that he had saved one case out of the nine, at the cost of absenting himself and several orderlies from the fighting-line for a fortnight or so. Clearly, from a military point of view, his course was not justified by results, and it is quite possible that the patient who recovered had only the semblance, not the reality, of a penetrating abdominal wound, and would have recovered in any event. At the base hospitals and clearing stations the mortality from abdominal wounds thus treated was terribly high —very much higher than in South Africa—and the results of the occasional surgical intervention which was tentatively adopted here and there were not encouraging.

* *Surgical Experiences in the South African Campaign.*

### Instant Laparotomy for Abdominal Wounds.

Within the last four or five months, however, a determined effort has been made to reduce this mortality by a trial of instant surgical intervention in the most favourable surroundings procurable. Several experienced operating surgeons have been posted to Clearing Stations, and arrangements have been made to facilitate and expedite the transfer of men believed to have wounds of the abdomen to those hospitals. A policy of exploratory laparotomy has been inaugurated, in place of the old method of rest and starvation; and, naturally, saline infusion has been largely resorted to for combating shock, a method practically in its infancy in 1900, and almost unknown in the hospitals of the South African Field Force. It is as yet, perhaps, too early for any final verdict as to the relative merits of the new system and the old one; but a valuable paper* by Colonel Cuthbert Wallace, a consulting surgeon to the Expeditionary Force, and in civilian life surgeon to St. Thomas's Hospital, makes out a *prima facie* case on behalf of laparotomy which demands the most respectful attention.

Colonel Wallace estimates the proportion of abdominal wounds to total wounds admitted to Field Ambulances as about 2 per cent.; the high mortality from such wounds results in a much lower percentage among the admissions to Clearing Stations. In the same way there is considerable difficulty in estimating the total mortality rate of abdominal wounds. Allowing for various disturbing factors, he places it at about 70 per cent., and he points out that owing to the nature of the injuries received the percentage of deaths must always be high. He lays it down that all cases should be operated upon early, if it be possible. "It is impossible within the period when operation will be most useful to distinguish between intra-abdominal hæmorrhage and an intestinal lesion complicated by hæmorrhage; nor is there any real need, as a man with a perforating wound of the abdomen should be operated upon on principle." If, however, the patient be not seen until twenty-four or thirty-six hours have elapsed since his wound, it is important to distinguish between peritonitis and hæmorrhage: "A patient hit through

* *Lancet*, December 18, 1915, p. 1336.

the abdomen thirty-six hours previously, and having a rigid and distended abdomen, is best left alone if he is in good condition and the pulse about eighty." Incidentally, the practice of exploration has confirmed what various surgeons had already suggested as possible—namely, that in a percentage of perforating wounds of the abdomen there is no injury to any important organ. A large proportion of such patients will recover without operation, and it is possible that to operate on them may slightly prejudice their chance of recovery. But there is no way of diagnosing between these cases and the cases of intestinal perforation *at a stage when operation will be of any benefit to the latter;* and Colonel Wallace is emphatic that many more lives are saved than are lost by early operation. None of the specialist operators working under him have any doubt, he says, as to the wisdom of the operative treatment. The only case of abdominal wound which has come under the writer's notice since the policy of exploratory laparotomy was adopted is one which well illustrates the success claimed by Colonel Wallace for that policy. An officer had a perforating abdominal wound, and was soon afterwards operated upon in a Clearing Station. Seven holes were found in his small intestines, and three in the large gut; he recovered practically without adverse symptoms. It is difficult to believe that this man can have had the smallest chance of his life if he had been treated on the expectant principle. Extensive statistics will be published in due course when a sufficiently large series of cases can be collected; meanwhile, we are well content to take Colonel Wallace's word for the benefits conferred on our troops by the opening of this new chapter in military surgery, and to congratulate him, his staff of surgeons, and the Army Medical Service in general on the results already and to be obtained.

The principles advocated by Colonel Wallace are further supported in an able *résumé* of clinical experience, written by Captain Meyer and Lieutenant Taylor, who have been working in a Casualty Clearing Station within the zone to which Sir A. Bowlby is consultant. In their first fifty cases of laparotomy for penetrating wound of the abdomen, seventeen died and the other thirty-three left the clearing station on a fair way to convalescence. Of forty-one operated upon during the same epoch, twenty-four were conservatively treated because it was thought from the clinical symptoms that recovery unaided was probable: in other words, it seemed likely that no mortal injury had been sustained. All the twenty-four confirmed this diagnosis by recovering. The other seventeen were not operated upon because their condition was deemed too desperate: all of them died. Thus it will be seen that these authors had fifty-seven recoveries (or presumed recoveries) out of ninety-one cases—truly a wonderful record.

